# Preoperative fluid restriction for trauma patients with hemorrhagic shock decreases ventilator days

**DOI:** 10.1002/ams2.328

**Published:** 2018-02-12

**Authors:** Shigenari Matsuyama, Ryusuke Miki, Hirotada Kittaka, Haruki Nakayama, Shota Kikuta, Satoshi Ishihara, Shinichi Nakayama

**Affiliations:** ^1^ Hyogo Emergency Medical Center Hyogo Japan

**Keywords:** Intensive care, outcome, prehospital care, resuscitation

## Abstract

**Aim:**

In recent years, with the concept of damage control resuscitation, hemostasis and preoperative fluid restriction have been carried out, but there is controversy regarding the effectiveness of fluid restriction.

**Methods:**

From April 2007 to March 2013, 101 trauma patients presented with hemorrhagic shock (systolic blood pressure ≤90 mmHg) at the prehospital or emergency department and were admitted to Hyogo Emergency Medical Center (Hyogo, Japan). They underwent emergency hemostasis by surgery and transcatheter arterial embolization. We compared two groups in a historical cohort study, the aggressive fluid resuscitation (AR) group, which included 59 cases treated in the period April 2007–March 2010, and the fluid restriction (FR) group, which included 42 cases treated in the period April 2010–March 2013.

**Results:**

There was no difference between both groups in patient background (heart rate, 110 b.p.m.; systolic blood pressure, 70 mmHg). The Injury Severity Score was 34 (AR) versus 38 (FR) (not significant). Preoperative infusion volume of crystalloid significantly decreased, from 2310 mL (AR) to 1025 mL (FR) (*P* ≤ 0.01). There was no difference in mortality (36% [AR] versus 41% [FR]). Ventilator days significantly decreased, from 8.5 days (AR) to 5.5 days (FR) (*P* = 0.02).

**Conclusions:**

Preoperative fluid restriction for trauma patients with hemorrhagic shock did not improve mortality, but it decreased ventilator days by reducing the perioperative plus water balance and it might contribute to perioperative intensive care.

## Introduction

Aggressive fluid resuscitation with rapid infusion of 1,000–2,000 mL crystalloid solution is widely used for diagnostic treatment of patients with hemorrhagic shock, as recommended by the Advanced Trauma Life Support (ATLS) guidelines. The addition of a prehospital medical transport system (ambulance or helicopter with physician) greatly expands the opportunities for aggressive fluid resuscitation.

Hyogo Emergency Medical Center (Hyogo, Japan) has operated an ambulance with physician service 365 days a year, 24 h a day, since it was established in 2003. Of approximately 400 annual cases, trauma accounts for the majority. For these patients, we initially actively undertook rapid crystalloid infusion to conform to the ATLS guidelines.

However, subsequent reports indicated negative outcomes with aggressive prehospital fluid resuscitation.^1^, ^2^, ^3^ We then changed the fluid strategy for hemorrhagic shock to preoperative fluid restriction using the massive transfusion protocol (MTP) introduced in 2010 in our center, and decreased the volume of preoperative fluid infused in prehospital and in‐hospital settings.

This study examined preoperative fluid restriction in severe injury cases with hemorrhagic shock to determine the effectiveness of this therapeutic strategy. This study was approved by the ethics committee of Hyogo Emergency Medical Center (ID: 2017009).

## Methods

Of 2,546 trauma patients transported to our center between April 2007 and March 2013, 2,109 were direct admissions. Transfer cases that were inappropriate for the preoperative infusion volume study were excluded. Four hundred and five cases had hemorrhagic shock and systolic blood pressure ≤90 mmHg at the time of initial evaluation. After excluding patients with cardiopulmonary arrest, 101 cases that underwent emergency surgery for hemostasis remained.

**Figure 1:**
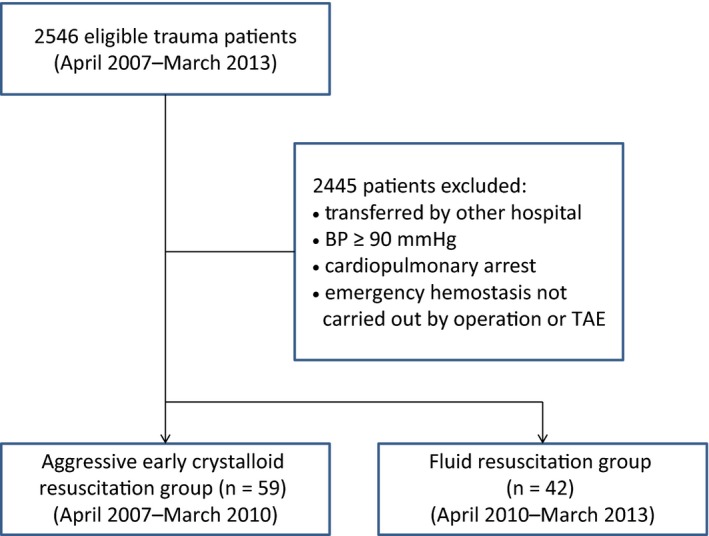
Flow diagram of the selection of our study population.

We divided patients into the aggressive resuscitation (AR) group (*n* = 59) transported between April 2007 and March 2010, when aggressive fluid resuscitation was enforced, and the fluid restriction (FR) group (*n* = 42) transported between April 2010 and March 2013, when MTP was introduced (Fig. 1). We did not establish rigid infusion volume or restriction guidelines, but instead decreased the infusion rate and withheld giving a rapid infusion if the systolic blood pressure was ≥80 mmHg, or the radial artery pulse was palpable. We compared the backgrounds of both groups, time to hemostasis, preoperative, intraoperative, and postoperative infusion and blood transfusion volumes for 24 h, death rates, ventilator days, and hospital days of survivors, using a historical cohort.

We defined time to hemostasis as time from contact with a patient to entry into the operating room or catheterization laboratory, and added transport time of ambulance with physician to the emergency department for spot sojourn time. The quantity of infusion, blood transfusion assumed it the last total dose in preoperation. In ambulance transport cases, heart rate and systolic blood pressure used the thing at the emergency department, and in the ambulance with physician transport cases with a thing at the time of the doctor contact at prehospital. Respiratory management methods did not change, and pressure control ventilation with SERVO‐i (MAQUET Holding B.V. & Co. KG, Rastatt, Germany) was used through both periods. Continuous variables were tested for normal distribution using the *F*‐test and examined with the Mann–Whitney *U*‐test. Qualitative variables were assessed using the χ^2^‐test for independence. Statistical analysis was carried out with Statcel3 software (http://www.oms-publ.co.jp/) and *P* < 0.05 was considered significant. Continuous variable data are shown as median and quartile values.

## Results

Age and sex were not significantly different between the two groups. The average heart rate was 110 b.p.m. and average systolic blood pressure was 70 mmHg in both groups. The primary cause of injury was blunt trauma in both groups. The Injury Severity Score (ISS) was 34 in the AR group and 38 in the FR group. Head injuries were present in 19% of the AR group and 24% of the FR group, showing no significant difference. Hemostasis was undertaken using surgery alone, surgery and transcatheter arterial embolization (TAE), or TAE alone, with no significant difference between groups (Table 1).

**Table 1:** Demographics and characteristics of 101 trauma patients with hemorrhagic shock treated with aggressive early crystalloid resuscitation (AR group) or fluid restriction (FR group)

	AR group (*n* = 59)	FR group (*n* = 42)	*P*‐value
Age, years	46 (31–65)	52 (41–66)	0.27
Male, %	64	69	0.63
Heart rate, b.p.m.	110 (80–126)	110 (82–129)	0.72
Systolic blood pressure, mmHg	70 (60–80)	70 (60–80)	0.43
Blunt injury, %	83	83	0.97
Injury severity score	34 (25–43)	38 (22–50)	0.51
Head injury, %	19	24	0.53
Hemostasis			0.95
Surgery	27	20	
Surgery + TAE	20	13	
TAE	12	9	

TAE, transcatheter arterial embolization, median (interquartile range).

**Figure 2:**
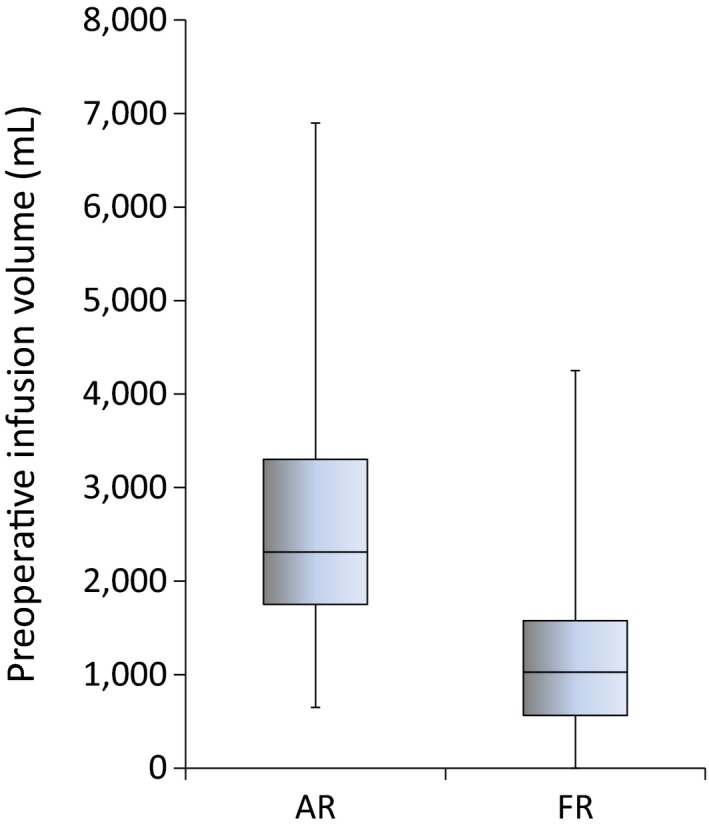
Preoperative infusion volume of crystalloid in 101 trauma patients with hemorrhagic shock treated with aggressive early crystalloid resuscitation (AR [*n* = 59]) or fluid restriction (FR [*n* = 42]).

The time to hemostasis in the FR group was 57 min compared to 77 min in the AR group. The preoperative infusion volume until hemostasis was 2,310 mL in the AR group and 1,025 mL in the FR group (Fig. 2). Only packed red blood cells were permitted for preoperative transfusion, with a maximum of 2 units; there was no significant difference between the groups.

**Figure 3:**
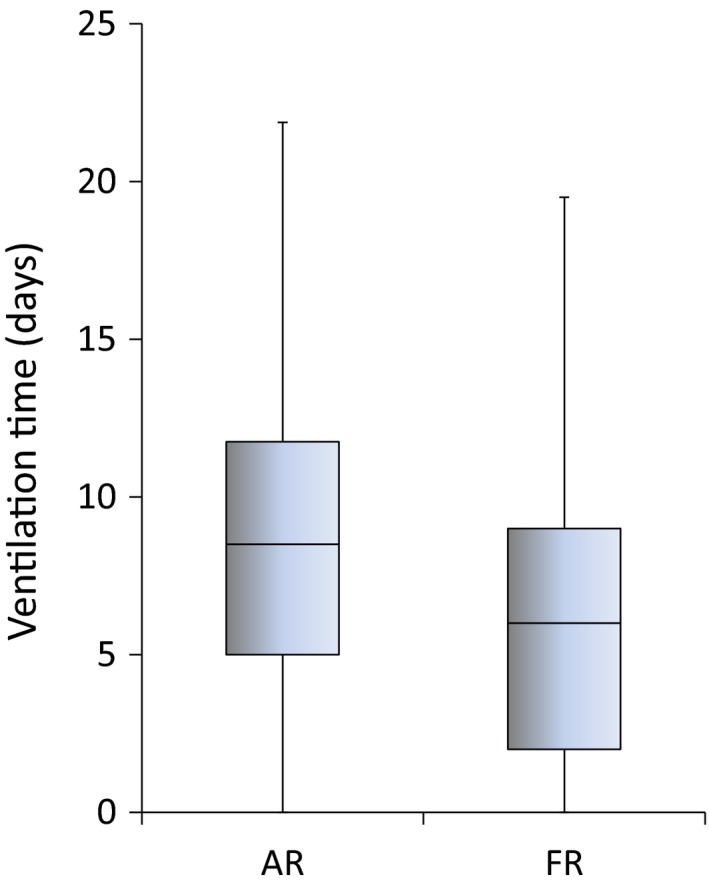
Number of required ventilator days in 63 survivors of trauma with hemorrhagic shock treated with aggressive early crystalloid resuscitation (AR [*n* = 38) or fluid restriction (FR [*n* = 25]).

The mortality rate was 36% in the AR group and 41% in the FR group, with no significant difference. There were 38 survivors in the AR group and 25 in the FR group. Mechanical ventilation was carried out for 6 days in the FR group survivors and 8.5 days in the AR group survivors (Fig. 3). The length of stay was 39 days in the FR group survivors and 34 days in the AR group survivors (Table 2). The intraoperative infusion volume was 2,535 mL in the AR group survivors and 1,350 mL in the FR group survivors. The 24‐h postoperative infusion volume was 5,273 mL in the AR group and 4,418 mL in the FR group, with no significant difference. The 24‐h postoperative blood transfusion volume was significantly decreased at 1,490 mL in the AR group and 440 mL in the FR group. The total intra‐ and postoperative infusion and blood transfusion volume was significantly decreased, with 14,708 mL in the AR group and 9,363 mL in the FR group (Table 3).

**Table 2:** Preoperative fluid, blood transfusion, time course, and outcome in 101 trauma patients with hemorrhagic shock treated with aggressive early crystalloid resuscitation (AR group) or fluid restriction (FR group)

	AR group	FR group	*P*‐value
Crystalloid, mL	2,310 (1,750–3,300)	1,025 (563–1,575)	<0.01
PRBC, units	2 (0–4)	2 (0–4)	0.54
Total time to hemostasis, min	77 (50–91)	57 (36–74)	0.03
Mortality rate, %	36	41	0.61
Ventilator days^a^	8.5 (5–12)	6 (2–9)	0.03
Hospital days^a^	34 (19–50)	39 (14–58)	0.81

a Survivors. AR group (*n* = 38) versus FR group (*n* = 25).

PRBC, packed red blood cells.

**Table 3:** Intraoperative and postoperative fluid and blood transfusion volumes in 63 survivors of trauma with hemorrhagic shock treated with aggressive early crystalloid resuscitation (AR group) or fluid restriction (FR group)

	AR group	FR group	*P*‐value
Intraoperative volume, mL			
Fluid infusion	2,535 (1,038–4,750)	1,350 (700–2,326)	0.04
Blood transfusion	2,600 (500–5,655)	1,840 (1,040–2,800)	0.26
Postoperative volume, mL (24 h)			
Fluid infusion	5,273 (3,881–7,320)	4,418 (3,849–6,766)	0.56
Blood transfusion	1,490 (590–3,290)	440 (0–1,080)	<0.01
Total in volume	1,4708 (8,712–19,581)	9,363 (6,459–12,253)	0.01

Median (interquartile range).

## Discussion

Our center specializes in managing severe emergency cases and receives 1,000 cases annually. Half of these are trauma patients, and half of these have severe trauma with an ISS ≥16. The injury observation cases during the study period included 2,546 cases, of which 101 cases of emergency hemostasis were the worst. The ISS for the AR group was 34 and that for the FR group was 38, with mortality rates of 36% and 41%, respectively. The ISS in a recent report that supports fluid restriction was 18–41, with a mortality rate of 9–30%,^4^, ^5^, ^6^, ^7^ so it can be considered that our cases were severe cases of the same or higher class.

In our strategy, we undertook aggressive fluid resuscitation in conformity with the ATLS™ guidelines from before hospitalization and gave ≥2,000 mL crystalloid during the approximately 30‐min transport in the ambulance with physician. It was not unusual to increase the quantity of fluid after arrival at the hospital. However, in recent years, negative reports on aggressive fluid resuscitation have been published. First, Bickell *et al*. reported that patients with body trunk penetrating trauma suffered shock before hospitalization. In that study, the patient group that underwent only securing of an i.v. line as much as possible, without transfusion, until operating room admission had a higher survival rate.^8^ Pepe *et al*.^2^ reported that fluid restriction should be carried out during the resuscitation of patients with hemorrhagic shock, before hemostasis, and they should be transported to the trauma center as soon as possible. Furthermore, Beekley proposed a new blood transfusion strategy of hemostatic resuscitation in addition to permissive hypotension for damage control resuscitation.^3^ In our center, a fluid strategy in the early period of trauma was reviewed for inclusion in the MTP introduced in 2010, and the prehospital infusion volume was significantly decreased.^9^

In this study, the infusion volume decreased in the FR group to 1,025 mL from 2,310 mL in the AR group due to the switch of strategy to preoperative fluid restriction, from patient contact to hemostasis before operation. The time to hemostasis from patient contact with the infusion volume was shortened to 57 min in the FR group from 77 min from the AR group. These are the total in‐hospital and prehospital times, which is the total of the spot stay and the transport time for the ambulance with physician to account for approximately 80% in both groups. The time to hemostasis from arrival was shortened from 53 min in the AR group to 42 min in the FR group. The time was shortened by approximately 20%, but the infusion volume decreased to 50% of that before operation and reflects the infusion speed in total. The early use of fresh‐frozen plasma and platelet concentrate was included in the protocol. In addition, emergency O‐type red blood cell concentrate from before operation by MTP introduction and the order of MTP from prehospital were enabled. However, a total red blood cell concentrate of 2 units was used only before the operation; the numbers of patients in both groups were less, and there was no difference in the quantity of blood transfusion.

The effectiveness of fluid restriction is still controversial. Yaghoubian *et al*.^10^ reported no significant difference in hospital mortality between the FR group and the non‐restricted group, even in patients with penetrating trauma. Brown *et al*.^4^ reported that the mortality of the non‐shock group increased in their study because of prehospital infusion of ≥500 mL, but reported no change in the shock group. The clinical effect of fluid restriction on perioperative management for non‐trauma patients is also controversial. Although some authors claim that, among patients who underwent gastrointestinal surgery, fluid restriction decreased complications and resulted in shorter hospital stay,^11^, ^12^ others denied its effect on hospital stay or morbidity,^13^ or even on complication rate.^14^

Morrison *et al*.^5^ reported that infusion and blood transfusion volume decreased because of fluid restriction, and that early mortality due to bleeding tendency improved. In a previous report, blood transfusion volume decreased because of fluid restriction even in cardiac surgery, during which mass transfusion was carried out.^15^ In our study, the infusion volume during hemostasis and the transfusion volume 24 h after surgery decreased in the FR group. The total infusion and blood transfusion volumes in the perioperative period decreased significantly. This suggests that preoperative infusion restriction facilitates hemostasis by preventing bleeding tendency, which is considered to contribute to decreased perioperative infusion and transfusion volumes. In addition, Kasotakis *et al*.^6^ reported that 24‐h crystalloid dose is related to duration of ventilator use and intensive care unit and hospital stay for fluid resuscitation of blunt trauma patients with hemorrhagic shock. In this study, ventilator days were shortened owing to the preoperative fluid restriction to compensate for the decrease in the perioperative plus water balance.

Duchesne *et al*.^7^ reported that not only was the 30‐day mortality improved due to fluid restriction and damage control resuscitation, including the MTP, but also the intensive care unit and hospital stays were shortened. However, in our study, the length of hospital stay was not shortened. Among the subjects of this study, 23 in the AR group and 16 in the FR group had a hospital stay of ≥30 days. Of these 39 cases, only 4 with single body trunk injury who were hospitalized because of digestive complications were converted and the other 35 had complicated injuries such as spinal, pelvic, limbs, or head injury. In these cases, because of the prolonged hospital stay due to delays in the improvement of performance in activities of daily living due to orthopedic trauma and delayed recovery from consciousness disturbance caused by head injury, we believe that the shortened intensive care period due to fluid restriction did not lead to shortened hospital stay.

As in previous reports, this study also supports fluid restriction, but the aggressive fluid resuscitation therapy has a diagnostic significance of estimating the bleeding rate by responding to rapid infusion. This study was limited to those subjects who received emergency hemostasis, and the FR group provided no opportunity to evaluate response to aggressive fluid resuscitation therapy. In fact, before experiencing fluid restriction, many patients attained natural hemostasis despite presenting with vital shock at the time of contact with a physician, responding to infusion and conservative treatment could be selected. However, we have experienced many cases in which patients who initially responded to rapid infusion experienced another drop in blood pressure after spending time undergoing computed tomography examination. Surgery and TAE are then hurried. From both experiences, before and after fluid restriction, we have found that hemostasis is more easily achieved when surgery is performed earlier. Therefore, cases certainly exist who presented with shock again after recovering circulation with aggressive fluid resuscitation therapy; in these circumstances, surgery is very difficult. For these patients, it is their last chance of survival when first responding to infusion. Although this study does not unconditionally support fluid restriction, in severe cases in which emergency hemostasis is inevitable, blood pressure temporarily increases because of aggressive fluid resuscitation therapy delays the judgment for performing emergency hemostasis, and hemostasis and perioperative management become difficult. We believe that fluid restriction is effective in these severe cases. Currently, aggressive fluid resuscitation therapy is regarded as a standard diagnostic treatment for judging the necessity of emergency hemostasis for active bleeding. However, in severe cases that require emergency hemostasis with effective fluid restriction, a further diagnostic method to judge the degree of active bleeding other than aggressive fluid resuscitation therapy should be established.

The limitations of this study are its single‐center retrospective design, the limited number of cases, and the differences in the period of occurrence of the cases.

## Conclusions

Although preoperative fluid restriction did not improve mortality in patients with hemorrhagic shock, the number of ventilator days were reduced by decreasing the perioperative plus water balance and contributing to acute intensive care.

## Disclosures

This study protocol was approved by the ethical committee of Hyogo Emergency Medical Center (ID: 2017009).

Informed consent was obtained from all patients.

Registration of the study/trial: N/A.

Animal studies: N/A.

Conflict of interest: None declared.
